# Node Detection Using High-Dimensional Fuzzy Parcellation Applied to the Insular Cortex

**DOI:** 10.1155/2016/1938292

**Published:** 2015-12-31

**Authors:** Ugo Vercelli, Matteo Diano, Tommaso Costa, Andrea Nani, Sergio Duca, Giuliano Geminiani, Alessandro Vercelli, Franco Cauda

**Affiliations:** ^1^GCS fMRI, Koelliker Hospital, Turin, Italy; ^2^Functional Neuroimaging and Neural Complex System Group, Department of Psychology, University of Turin, Turin, Italy; ^3^Department of Psychology, University of Turin, Turin, Italy; ^4^Neuroscience Institute of the Cavalieri Ottolenghi Foundation and Department of Neuroscience, University of Turin, Turin, Italy

## Abstract

Several functional connectivity approaches require the definition of a set of regions of interest (ROIs) that act as network nodes. Different methods have been developed to define these nodes and to derive their functional and effective connections, most of which are rather complex. Here we aim to propose a relatively simple “one-step” border detection and ROI estimation procedure employing the fuzzy *c*-mean clustering algorithm. To test this procedure and to explore insular connectivity beyond the two/three-region model currently proposed in the literature, we parcellated the insular cortex of 20 healthy right-handed volunteers scanned in a resting state. By employing a high-dimensional functional connectivity-based clustering process, we confirmed the two patterns of connectivity previously described. This method revealed a complex pattern of functional connectivity where the two previously detected insular clusters are subdivided into several other networks, some of which are not commonly associated with the insular cortex, such as the default mode network and parts of the dorsal attentional network. Furthermore, the detection of nodes was reliable, as demonstrated by the confirmative analysis performed on a replication group of subjects.

## 1. Introduction

One powerful method for studying brain organization is the graph-theoretical approach [1]. Similar to other connectivity methods, such as seed-based functional connectivity and diffusion-tensor-imaging approaches, this method requires the definition of a set of regions of interest (ROIs) that act as network nodes [2]. Several techniques have been employed to functionally derive these nodes and their connections [3, 4]. The definition of such nodes often involves complicated functional connectivity estimation and border detection procedures [5]. Here, we suggest a relatively simple “one-step” border detection and ROI estimation procedure. In particular, we propose to take advantage of one of the characteristics of the fuzzy *c*-mean clustering algorithm [6]. This procedure allows a fixed percentage of voxels with a borderline pattern of connectivity to be nonunequivocally attributed. The further we move from the centre towards the border of a cluster, the more the characteristics of the pattern of connectivity are intermixed with those of the neighbouring clusters (e.g., nonunequivocally determined). As was recently shown by Smith et al. [7], the maximization of spatial independence could lead to suboptimal detection of networks that share significant spatial overlaps. Our method maximizes the temporal independence because the criterion used in the clustering algorithm is the correlation between the time series of each voxel and the time series of the centre of each cluster.

Previous clustering studies [6, 8–10] performed the clustering procedure at subject level. Given the relative deficiency of time points (about 120–200 points in a six-minute run), this procedure has good reliability only for low-dimensional parcellation (e.g., with a limited number of clusters). In line with other investigations [2, 11, 12], we concatenated the time courses across all subjects to constitute a very big dataset that allowed us to obtain a higher clustering dimensionality. Far from representing a step backwards, this “fixed-effect” approach permits good estimation of a common set of clusters (and thereby of nodes) for a given group of subjects. Subsequently, the between-subject variance was taken into consideration, so that each node's functional connectivity pattern was evaluated at the subject level and summarized using a random-effect analysis. This method is very similar to the dual regression approach [13], according to which the independent component analysis (ICA) was first applied to the concatenated dataset.

To investigate how this node detection method performs with real data, we applied our procedure to the insular surface of 20 healthy subjects scanned in a resting state. A second dataset of 18 healthy volunteers was used for replication testing. We chose the insular surface because the insula is a complex and pivotal [14] brain area in which different inputs from the body and the external world are highly integrated [15]. This brain region has been parcellated by using different measures, such as resting-state functional connectivity [8, 10, 14], task-related functional connectivity [16, 17], and diffusion tensor imaging [18, 19], into two [8, 16, 20–23], or three [9, 10], or more clusters [4, 5, 17, 24], each of which has a unique pattern of connectivity. A recent paper by Kelly et al. [25] demonstrated a convergence between resting state, task-based functional connectivity, and anatomical coactivations at several different parcellation levels (from two to 12 clusters per side; with more than 12 clusters, reliability dropped by about 50%), thus supporting a common hierarchical structure within the insular cortex.

Given these premises, it would be of great interest to test this new node detection method in search of a resting-state functional parcellation of the insular surface. As an additional consideration, we suppose that high-dimensional clustering [11] can make it possible to demonstrate the existence of a more complex pattern with “echoes” [12] of several brain networks nested within the two main insular patterns previously reported.

## 2. Methods

### 2.1. Main Group

Main group consists of twenty healthy right-handed volunteers (10 females, with a mean age of 32.6 ± 11.2). Replication group comprises eighteen healthy right-handed volunteers (nine females, with a mean age of 25.3 ± 4.2). All subjects were free of neurological or psychiatric conditions, were not taking any medication known to alter brain activity, and had no history of drug or alcohol abuse. Handedness was ascertained with the Edinburgh Inventory [26]. We obtained written informed consent from every subject, in accordance with the Declaration of Helsinki. The study was approved by the institutional committee on ethical use of human subjects at the University of Turin.

### 2.2. Task and Image Acquisition

Images were acquired during a resting-state scan on a 1.5 Tesla INTERA scanner (Philips Medical Systems). Functional T2^*∗*^ weighted images were acquired using echoplanar (EPI) sequences, with a repetition time (TR) of 2000 ms, an echo time (TE) of 50 ms, and a 90° flip angle. The acquisition matrix was 64 × 64, with a 200 mm field of view (FoV). A total of 200 volumes were acquired, with each volume consisting of 19 axial slices; slice thickness was 4.5 mm with a 0.5 mm gap, while in-plane resolution was 3.1 mm. Two scans were added at the beginning of functional scanning to achieve steady-state magnetization before acquiring the experimental data. A set of three-dimensional high-resolution T_1_-weighted structural images was acquired, using a fast field echo (FFE) sequence, with a 25 ms TR, an ultrashort TE, and a 30° flip angle. The acquisition matrix was 256 × 256 and the FoV was 256 mm. The set consisted of 160 contiguous sagittal images covering the whole brain.

### 2.3. Data Analysis

Datasets were preprocessed and analysed using BrainVoyager QX software (Brain Innovation, Maastricht, The Netherlands).

Functional images were preprocessed to reduce artefacts as follows [27]: (i) slice scan time correction was performed using a sinc interpolation algorithm; (ii) 3D motion correction was applied using a trilinear interpolation algorithm according to which all volumes were spatially aligned to the first volume by rigid body transformations, and the roto-translation information was saved for subsequent elaborations; (iii) spatial smoothing was performed using a Gaussian kernel of 8 mm FWHM. Several nuisance covariations were regressed out from the time courses to control for the effects of physiological processes, such as fluctuations related to cardiac and respiratory cycles and motion [28–30]. Specifically, we included nine additional covariations from white matter (WM), global signal (GS) [31], and cerebrospinal fluid (CSF), as well as six motion parameters. Subsequently, time courses were temporally filtered in order to keep only frequencies between 0.008 and 0.08 Hz and normalized.

Following the preprocessing, we implemented some steps to improve intersubject analysis of the anatomical location of brain activations. For each subject the functional scans were coregistered with a relatively high-resolution structural scan. This coregistration was done using both the slice positioning as stored in the raw data's headers and fine adjustments calculated comparing the intensity values of the data sets. After this we transformed each subject's 3D structural data into Talairach space [32]. This was obtained by translating and rotating the cerebrum on the plain passing through the anterior and the posterior commissure; then, the borders of the cerebrum were identified. The coregistration matrix of anatomical and functional data consisted of the parameters of rotation and translation during the coregistration step and the parameters of Talairach normalization. Finally, by applying the anatomical-functional coregistration matrix we transformed into Talairach space the functional time course of each subject and created the volume time course.

We applied a fuzzy *c*-mean algorithm to the time courses of all the insular voxels and clustered these voxels on the basis of their temporal similarity. As is typical of fuzzy clustering techniques, a certain percentage of voxels can be nonunivocally attributed to the parcels. The percentage of nonunivocally attributed voxels (the fuzziness coefficient) is an arbitrary parameter. In line with other studies [33], we chose 20% of nonunivocally attributed voxels.

The fuzzy clustering technique parcels out a subset of *N* voxels in *C* “clusters” of activation [34]. Signal time courses of all voxels were* z*-standardized. We subsequently confronted the voxel's time courses **x**_*n*_ (*n* = 1 ⋯ *N*) with each other and derived a representative cluster of time courses (cluster centroids) **v**_*c*_ (*c* = 1 ⋯ *C*). On the basis of this unsupervised method, and starting from the original fMRI time series, we got a predefined number of spatial modes, which were composed of a spatial map and an associated centroid time course. Accordingly, a voxel is assigned to a cluster with reference to the similarity (e.g., by correlation) of its time course to the cluster centroid. This similarity is determined in a fuzzy way, which means that a voxel is not uniquely assigned to one cluster (hard clustering) because the similarity is expressed by the “membership” *u*_*cn*_ of voxel *n* to cluster *c*.

Centroids **v**_*c*_ and memberships *u*_*cn*_ are both updated in an iterative procedure [35], which terminates when successive iterations do not further change memberships. Cluster centres are determined via classical cluster-algorithm distance measures and are expressed as follows:
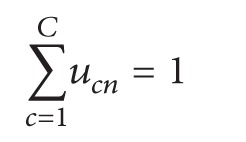
(1)

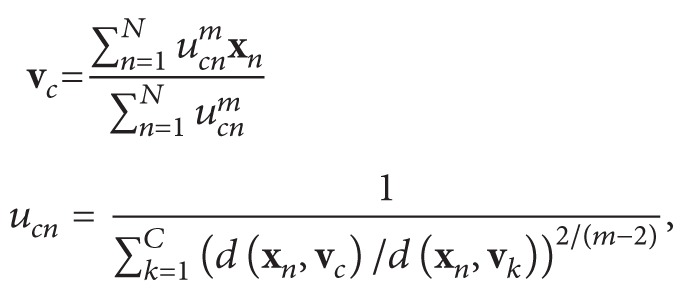
(2)where *d* is the distance between a voxel and a cluster centre and *m* is the coefficient that determines the fuzziness of the procedure; *m* “tunes out” the noise in the data and lies between 1 (smallest fuzziness) and infinity. The most commonly used of the several distance measures of *d* are the Euclidean distance, *d*_*E*_, and the Mahalanobis distance, *d*_*M*_ [36], which are defined as

(3) dExn,vc=xn−vc2,dMxn,vc=xn−vcTΣc−1xn−vc.

Σ_*c*_ is the covariance matrix of cluster *c*. The Mahalanobis distance takes in the elliptical shape of the cluster (i.e., it weights the differences by the range of variability, described by Σ_*c*_, in the direction of the voxel instead of treating all voxels **x**_*n*_ equally when calculating the distance *d* to the cluster centre **v**_*c*_). The Euclidean distance assumes a spherical shape, without taking into account the shape of clustering, which corresponds to a covariance matrix Σ_*c*_ with 1 s on the main diagonal and 0 s elsewhere.

Calculation starts from an initial set of membership values for the data set in the following matrix form:

(4) $$\begin{array}{c} U^{\left(0\right)}=\left(\;1-\frac{\sqrt{2}}{2}\;\right)U+\frac{\sqrt{2}}{2}V \end{array}$$

with *U* = 1/*C* and *V* a matrix of randomly chosen cluster centres with initial *C* = 2. Subsequently, the new centroids and memberships are calculated using (2). When further iterations do not cause significant change to memberships and centroids, the procedure stops. With this procedure the following function is minimized:

(5) $$\begin{array}{c} \sigma_{w}^{2}=\frac{1}{N}\overset{N}{\underset{n=1}{\sum }}\;\overset{C}{\underset{c=1}{\sum }}u_{cn}^{m}d\left(\;x_{n},v_{c}\;\right). \end{array}$$

This formula calculates the within-class variance over all clusters, *σ*_*w*_^2^. In other words, a user-defined threshold for change in *σ*_*w*_^2^ is fixed when convergence is reached. The criterion of convergence is based on the first local optimum of (5) [37]. Each time series is transformed into its *z*-score in order to avoid a classification of the voxels on signal amplitude rather than on signal shape. Then, the principal component analysis (PCA) is performed to reduce data dimensionality. The number of PCA components was calculated to retain 95% of the variance.

Optimal number of clusters: The a priori definition of the number of clusters and the fuzziness coefficient is often debated in the literature [38]. Usually, the optimal number of classes is unknown in fuzzy clustering. A number of cluster-validity indices have been proposed to estimate the optimal number of clusters in an unsupervised manner (for a review see [39]). These indices aim to identify compact and well-separated clusters. In this study, we used the silhouette validation method [40], which consists in considering the silhouette coefficient of each element:

(6) $$\begin{array}{c} s_{i}=\frac{b_{i}-a_{i}}{\max\left(b_{i},a_{i}\right)}, \end{array}$$

where *a*_*i*_ is the average dissimilarity of the *i*-point to all points in the same cluster and *b*_*i*_ is the minimum of the average dissimilarity of the *i*-point to all points in the other cluster.

Unlike Cauda et al. [8], to perform the clustering procedure time courses were concatenated across all subjects; this step, as has been pointed out by others [2], makes it possible to obtain a higher clustering dimensionality (i.e., more clusters).

Due to the fuzziness coefficient employed, 20% of voxels were classified as nonunequivocal. We considered these voxels as border voxels, with a time course that showed transitional characteristics between contiguous clusters.

**Figure 1:**
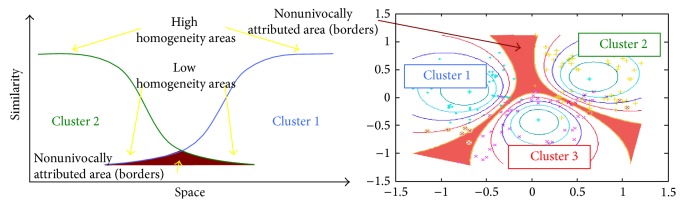
Infographic depicting the functioning of the method.

The final step of this procedure was to place a spherical ROI with a radius of 3 mm in the local maxima of each cluster (i.e., the area of maximal similarity between voxel time courses). See Figure 1 for a graphical representation of the method.

To investigate the specific pattern of connectivity of each cluster we employed a variant of the dual regression approach [13]. In brief, we performed a generalized linear model (GLM) including all the subject-specific time courses of all 12 right-insular spherical ROIs in a multiple regression analysis. This method resulted in a subject-specific time course relative to each ROI, while controlling for the variance explained by all the other ROIs [12]. Subject-specific patterns of functional connectivity relative to each ROI were then summarized at a group-level using a one-sample *t*-test.

**Figure 2:**
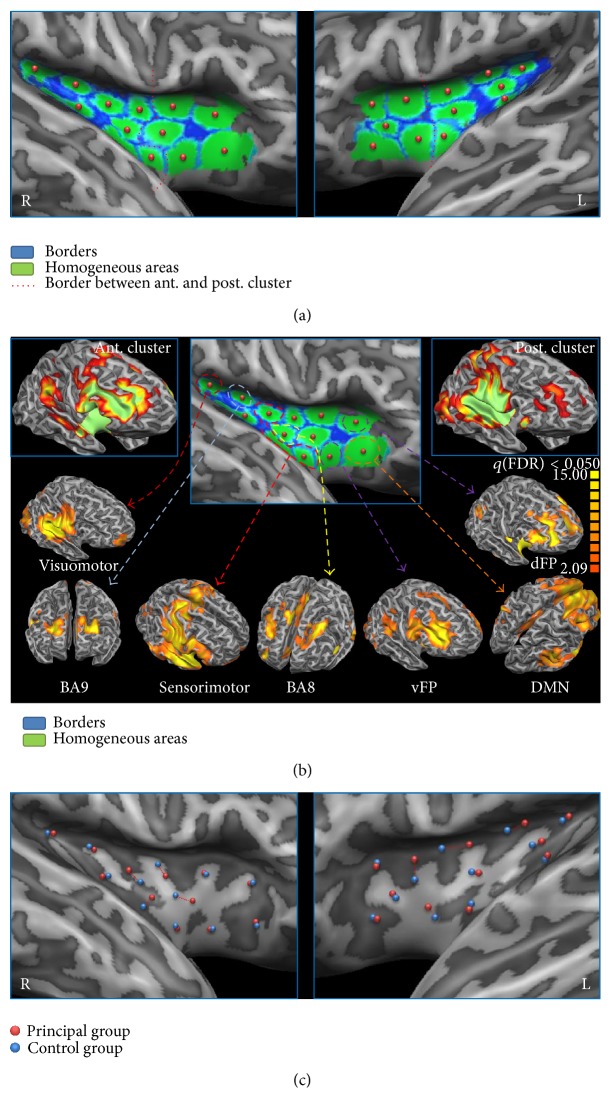
High-dimensional clusterization and node creation of the insular cortex. (a) High-dimensional insular clusterization. Homogeneous areas are shown in green and borders in blue. The dotted red line outlines the separation between anterior and posterior clusters as detected in our previous studies [8, 33]. (b) Upper panels: anterior- and posterior-cluster functional connectivity. (b) Lower panels: patterns of functional connectivity regressing out the common variance. (c) Reliability of node creation. Nodes calculated from the main group are shown in red, nodes calculated from the replication group in blue. If separated, paired nodes are shown by a dotted red line.

To confirm previous connectivity results [8], we also calculated the functional connectivity of the anterior and posterior insular clusters by grouping together the ROIs belonging to each cluster (see Figure 2(a)).

To validate our parcellation results we applied our method to a second dataset (replication group) and compared the results of the two datasets.

All maps were thresholded at *p* < 0.05 and corrected for multiple comparisons using the false discovery rate (FDR).

## 3. Results

Our method was able to separate 12 clusters for each insula. This number turned out to be the preferred number of clusters after the application of the silhouette validation method [40]. The algorithm returned the borders of the functionally homogeneous areas; for each cluster a spherical ROI with a radius of 3 mm was placed in the area with the maximal homogeneity (see Figure 2(a)). These results were replicable. Indeed, as shown in Figure 2(c), all the ROIs were also found in the control group and the locations were almost overlapping in 17 out of 24 ROIs, while the other ROIs were displaced by only a few millimetres.

Our calculation of the functional connectivity of the ROIs belonging to anterior and posterior insular clusters confirmed the two patterns of connectivity described in a previous study [8]. The anterior pattern, which occupies the most anterior ventral part of the insular cortex, is characterized by a cingulate-frontoparietal connectivity that has often been related to salience detection. In turn, the posterior pattern, which occupies the posterior dorsal insula, principally shows a sensorimotor connectivity pattern. However, by considering all the 12 ROIs and including the ROI time courses in a multiple regression analysis [12], we discovered a much more intricate picture. With this method we demonstrated that these areas are connected to several other networks, such as the default mode network, the sensorimotor network, and parts of the dorsal attentional network.

## 4. Discussion

By focusing on resting-state data, this study has demonstrated that the proposed fuzzy clustering node detection approach is able to perform simple yet reliable node detection and surface parcellation.

By virtue of the fuzzy clustering procedure, we successfully generated nodes, and the data obtained with this method were replicable with other datasets. This method allows a very simple one-step border detection procedure, taking advantage of the fact that the borders between areas with homologous functional connectivity are characterized by a temporal profile (i.e., time course) with mixed characteristics of time courses of contiguous regions. These time courses were therefore nonunivocally classified.

The fuzzy clustering procedure has proven to be replicable using a replication dataset. By varying the fuzziness coefficient it is possible to change the number of voxels that are attributed to borders, and as a consequence the homogeneity of the voxels pertaining to the univocally defined parcels. Furthermore, by applying a high-dimensional clustering procedure to the analysis of the functional connectivity of the insula, we were able to detect the connectivity patterns, or, as defined by Leech et al. [12], the “echoes” of the other neural networks that we hypothesized might constitute the hierarchical subparcellation of the aforementioned anterior and posterior patterns of connectivity: the ventral anterior cingulo-fronto-parietal “salience detection” network and the dorsal posterior sensorimotor network, respectively.

As has been previously reported [8, 16, 25, 33], there was some interhemispheric lateralization in the connectivity patterns of the insula. Indeed, the localization of the clusters in the right and the left insula was slightly different, especially in the posterior insula. These results were confirmed when we subdivided the ROIs on the basis of their involvement within the two anterior and posterior clusters. A possible explanation for this hemispheric asymmetry could involve some aspects of emotional and sympathetic processing; for example, the right insula is likely to respond more to sympathetic arousal, and the left insula to parasympathetic nervous functions [41, 42]. Furthermore, the anatomical connections of the anterior insula (AI) with areas pertaining to the ventral attentional network, in particular the temporoparietal junction (TPJ), have been shown to be lateralized on the right side [43]. This is coherent with the “fight or flight” function of the sympathetic system, which requires an evaluation of the potential danger of incoming stimuli.

The posterior part of the insula is characterized by smaller clusters and less homogeneity than the anterior part. Accordingly, hemispheric asymmetry is also more evident in the posterior than in the AI. These findings support the idea that the posterior circuits have more heterogeneous connectivity patterns, in line with Craig's hypothesis suggesting that the posterior insula is a sort of data collector linked to many different networks [15, 44]. As reported in recent studies [9, 25], this group of posterior clusters mainly exhibits a sensorimotor pattern of connectivity. Indeed, if we move from the more posterior part of the insular surface towards the middle and anterior parts, the connectivity changes from visuomotor to prefrontal pathways (BA9), then to sensorimotor, and back again to prefrontal pathways (BA8) within the middle insular cortex. In fact, not only action and perception-related patterns but also some prefrontal patterns of connectivity are present within the posterior insula. This is probably due to the involvement of this area in a variety of different activities, such as pain, language, interoception, and sexuality, as has been recently reported [17, 25]. Overall, the posterior insula shows a more specific connectivity pattern than the AI, which, on the contrary, shows connections with networks related to the switch of attention between internal and external stimuli, such as the attentional and default mode networks. Thus, rather than being specific, this pattern suggests a general function that can be exploited in a variety of everyday activities. This has been confirmed by several papers that have shown how the AI is more aspecifically and massively activated in a broad series of different behavioural domains [8, 9, 17, 25]. In line with our data, these studies linked the activity of the AI with cognitive and emotional responses, an involvement that, together with saliency detection [45] and task switching [46], is almost ubiquitous.

Some authors [10, 14, 17, 47–49] have suggested a differentiation or a gradient of connectivity between the dorsal and ventral AI, a variance that, however, we failed to demonstrate in our previous papers. Different levels of parcellation determined by the various methods used to calculate the optimal number of clusters lead to a different picture of the insular cortex. This phenomenon is particularly evident in the results of the study by Kelly et al. [25], which compared insular parcellations with *n* = 2 and *n* = 3 clusters. In the parcellation with *n* = 3 clusters the higher number of clusters made it possible to reveal an anterior ventral cluster that was not present with *n* = 2.

In the present paper the complex structure of the anterior insular cortex has been further clarified. We have validated the recent identification made by Touroutoglou et al. [50] of two dissociable frontoparietal patterns of functional connectivity, the dorsal and ventral AI, respectively (for a similar result see also [14]). These two networks (the dorsal one here referred to as dFP and the ventral one as vFP) probably subserve only two partially different functions. The dorsal network is likely to be more involved in the integration of top-down and bottom-up salient information, whereas the ventral network is likely to be more involved in aspects of emotional salience detection as well as the integration of bodily feelings [50]. These two large-scale networks exhibit a different pattern of connectivity: the dorsal network is more centred on dorsolateral and dorsomedial prefrontal cortices plus mid-dorsal cingulate cortices, whereas the ventral network is more linked to the anterior cingulate, ventral prefrontal cortices, and TPJ. Other authors have identified a network that is similar to the dorsal anterior cluster, or, rather, to a mix of the dorsal and ventral anterior insular clusters, for example, the frontoparietal control network [51], and the cingulo-opercular, ventral attentional [52, 53], and basal ganglia-fronto-insular [53] control networks [14, 46]. The two anterior insular frontoparietal networks show areas of overlap and might have a shared variance that in some conditions makes these two components less easily separable.

Interestingly, a cluster placed in between and just anterior to these two areas shows connectivity with the default mode network. This cluster resides in a position that largely overlaps with the agranular area described by Marsel Mesulam and Mufson in 1982 [54]. This result seems to validate the hypothesis according to which the AI is placed in a pivotal brain site so as to continuously reallocate cerebral resources between internal and external focused networks [55, 56] and modulate the switch between goal-oriented attentional and default mode networks. This supposition would also explain the frequent activation of this brain area in so many different tasks.

## 5. Conclusions

The dFP and vFP patterns of connectivity, which further subdivide the anterior and posterior insular clusters, are probably overcome by the variance of two other main patterns, but when this variance is regressed out, a more complex picture emerges. This phenomenon can be explained by the hierarchical connectivity structure of the insular cortex, as has been suggested by some authors [16, 25]. The two clusters that were previously identified in our papers were here divided into a series of smaller parcels, a procedure in line with recent studies [18, 25, 57]. This is also in accordance with the suggested intrinsically hierarchical structure of this area [5, 24, 58], as hypothesized by Craig and Damasio [15, 41, 59].

In this study we were able to demonstrate that the detection of nodes using high-dimensional fuzzy *c*-mean parcellation is a simple, efficient, and reliable method. This indicates that the insula displays a potentially hierarchical structure, in which information coming from the environment and from the body is integrated and distributed to different areas of the brain. Although our study confirmed the two (or three) major insular subdivisions, a more in-depth investigation also showed that these areas can be further subdivided into smaller clusters, each characterized by its own pattern of connectivity that can be detected with appropriate techniques.
